# Information in Spanish on the Internet about the Prevention of COVID-19

**DOI:** 10.3390/ijerph17218228

**Published:** 2020-11-07

**Authors:** Ignacio Hernández-García, Teresa Giménez-Júlvez

**Affiliations:** 1 Department of Preventive Medicine and Public Health, Lozano Blesa University Clinical Hospital of Zaragoza, Calle San Juan Bosco 15, 50009 Zaragoza, Spain; 2 Department of Preventive Medicine and Public Health, Miguel Servet University Hospital of Zaragoza, Paseo Isabel la Católica 1, 50009 Zaragoza, Spain; tgimenez@salud.aragon.es

**Keywords:** COVID-19, coronavirus, internet, information, prevention, World Health Organization, Spanish

## Abstract

*Objective*. Our objective was to analyze the evolution of the information in Spanish online about the prevention of the coronavirus disease 2019 (COVID-19). *Methods*. On 1 March and 13 July 2020, two searches were conducted on Google with the terms “Prevencion COVID-19” and “Prevencion Coronavirus”. In each stage, a univariate analysis was performed to study the association of the authorship and country of origin with the basic recommendations to avoid COVID-19 provided by the World Health Organization (WHO). *Results*. A total of 120 weblinks were evaluated. The recommendation found most frequently in both stages was “wash your hands frequently” (93.3% in March vs. 90.0% in July). There was a significant increase in the detection of the following recommendations: “avoid touching your face” (56.7% vs. 80.0%) and “stay at home if you feel unwell” (28.3% vs. 63.3%). Weblinks of official public health organizations more frequently provided the advice to “seek medical advice if you develop a fever/cough or have difficulty breathing”. Furthermore, in July, such weblinks provided recommendations to “avoid touching your face” and “maintain a distance of one meter” more frequently than the mass media (OR = 11.5 and 10.5, respectively). In March, the recommendation to “maintain a distance of at least 1 m” was associated with the weblinks from countries with local transmission/imported cases (OR = 8.1). Different/ambiguous information regarding the WHO recommendations was detected in four weblinks. *Conclusion*. The availability of information in Spanish online on basic prevention measures has improved over time, although there is still room for improvement. It is necessary to promote the use of the websites of official public health organizations among Spanish-speaking users.

## 1. Introduction

In December 2019, an outbreak of the coronavirus disease 2019 (COVID-19), which is caused by the novel severe acute respiratory syndrome coronavirus 2 (SARS-CoV-2), emerged in the city of Wuhan (Hubei province, China). On 30 January 2020, the World Health Organization (WHO) declared the outbreak a Public Health Emergency of International Concern [1]. Subsequently, on 11 March 2020, the WHO recognized it as a global pandemic [2].

According to the WHO situation reports, on 13 July 2020, the virus had infected 1,638,378 people in Spanish-speaking countries, causing 99,904 deaths; Peru, Chile, Mexico, and Spain had the highest number of infected people, with more than 250,000 cases in each country, while Mexico and Spain had the highest number of deaths, with 34,730 and 28,403, respectively [3].

In this context, the presidents of several governments (such as those of Chile, Peru, and Colombia) have stated that this pandemic has taught them that their priority must be education [4]. In particular, educating the population about basic measures to prevent COVID-19 is especially important since there is no specific treatment or vaccine [5].

The Internet can play a very important role as an educational tool, since it is the largest and fastest source of health information [6], and it has the capacity to influence its users [7]. In particular, health information on websites is increasingly taken into account by their users when making decisions about health care [8,9]. In some countries, such as the United States of America, 59% of adults look for health information on websites [10], 6.75 million health-care-related searches are performed per day [11], and 35% of people use web-based health information to make diagnoses, but only half of them check with medical professionals [10].

Information on the Internet, if evidence-based and unbiased, can improve people’s health knowledge and assist them in disease management [12]. In the last ten years, the availability of health information online has drastically increased [13]. However, it often lacks scientific rigor, as anyone may upload content. This fact is of great concern to scientific societies, governments, and users [14].

In the recent Ebola [15] and Zika [16] epidemics, as well as in the current COVID-19 pandemic, the Internet has been used as a means for the spread of information (and misinformation) [15,16]. It has implications for health-related decision-making and public health behavior because people want to know what they can do to prevent the disease [17]. For this reason, it is necessary for health organizations to quickly respond to and correct the information on the Internet in order to gain the public’s trust and influence it to follow their recommendations [17]. In fact, the fight against the COVID-19 pandemic is also the fight against an “infodemic” [18].

An infodemic is an overabundance of information—some accurate and some not—occurring during an epidemic. An infodemic makes it hard for people to find trustworthy sources and reliable guidance when they need it [19]. Infodemiology is the science of managing infodemics [19]. In this context, infodemiological studies are becoming increasingly necessary, since they can provide valuable insights into health-related behaviors of populations. Infodemiology studies the distribution and the determinants of information on the Internet or in a population, and aims to inform public health and public policy [20]. Examples of infodemiology applications include the identification and monitoring of public-health-relevant publications on the Internet, measuring information diffusion, and analyzing how people search and navigate on the Internet for health-related information as well as how they communicate and share this information [20,21].

Numerous studies have analyzed the information available on the Internet on the epidemiology, diagnosis, and treatment of COVID-19 [22,23,24]. However, few studies have evaluated the information currently available online on how to prevent it, despite the importance of these measures given that there is currently no vaccine or specific antiviral treatment. Such studies, specifically those conducted in the early stages of the pandemic, noted that there was a difficulty in finding information on the basic prevention measures recommended by the WHO to control it [25].

This research was carried out with the aim of evaluating the evolution of the spread of the basic prevention measures of COVID-19 recommended by the WHO, available in Spanish on the Internet, and associated factors.

## 2. Materials and Methods

We performed a repeated cross-sectional observational study on the Internet. On 1 March 2020 and 13 July 2020, from a Spanish IP address, two Google searches were performed with the terms “Prevencion COVID-19” and “Prevencion Coronavirus”. Like in other studies, the first 30 weblinks in Spanish were selected for each search, excluding advertisements [26,27,28]. While using Google, the author was not logged in as a Google user. The search history and cookies were not cleared before the new second search.

The information corresponding to the following variables was extracted from each weblink: type of authorship (official public health organization, official non-public-health organization, scientific society, digital mass media, library, private health care system, etc.), country of origin, and basic protective measures to avoid COVID-19, according to the WHO, effective at the time of the searches, including (a) regularly and thoroughly cleaning one’s hands; (b) maintaining a distance of at least 1 m (3 feet) between oneself and others; (c) not touching the eyes, nose, and mouth; (d) covering one’s nose and mouth with a bent elbow or tissue if one is sneezing or coughing (then disposing of the used tissue immediately); (e) staying at home if one feels unwell; and (f) if one develops a fever/cough or has difficulty breathing, seeking medical advice promptly (calling in advance) [29]. The information was obtained by making up to four clicks on the different sub-links of each link, as has been done in other studies [25,30].

A Chi-squared test was conducted to study the association between the date of the search (1 March 2020, or 13 July 2020) and the frequency with which the basic prevention measures were found. The associations of the variables of type of authorship and country of origin with the detection of basic prevention measures were also analyzed using a Chi-squared test or Fisher’s exact test for each search date. For this purpose, the variable of type of authorship was categorized as follows: official public health organization, digital mass media, etc. The countries of origin of the weblinks were categorized according to the type of transmission existing in each country at the time of the data collection and were as follows: (a) no transmission: countries where no cases were reported; (b) imported cases only: countries where all cases were acquired outside the reporting location; (c) local transmission: countries where the source of infection was within the reporting location; (d) clusters of cases: experiencing cases clustered in time, by geographic location, and/or by common exposures; and (e) community transmission: experiencing larger outbreaks of local transmission defined through an assessment of factors, including, but not limited to, large numbers of cases not linkable to transmission chains, large numbers of cases from sentinel lab surveillance, and/or multiple unrelated clusters in several areas of the country [3,31].

The strength of the association was quantified with the Odds Ratio (OR) and its 95% confidence interval (95% CI) obtained from univariate logistic regression analysis. In all hypothesis tests, differences with a *p* value of <0.05 were considered statistically significant. All analyses were performed using Epi Info^TM^ (Centers for Disease Control and Prevention, Atlanta, GA, USA) and SPSS v25 (IBM Corp, Chicago, IL, USA). As in other studies, the information available on the Internet was assessed, and no human participants or animals were included. For this reason, ethical approval was not required [6].

## 3. Results

In total, 120 weblinks were reviewed. Most of them were produced in Spain (70.0% (84/120)) by official public health organizations and digital mass media (60.0%) (Table 1). Globally, throughout the study, the WHO’s preventive measures mentioned most often were “wash your hands frequently” (91.7%) and “cover your mouth and nose when you cough or sneeze” (82.5%). A recommendation less frequently made was to “stay at home if you feel unwell” (45.8%) (Table 2).

According to the search date, on 13 July 2020, a significant increase was observed in the number of weblinks produced from Spain (Table 1). Likewise, a significant increase in the number of weblinks produced by official public health organizations was detected, as well as a significant decrease in the number of weblinks from digital mass media (Table 1).

Over time, the number of weblinks that provided information on some measures to prevent COVID-19 (“avoid touching your eyes, nose, and mouth”, and “stay at home if you feel unwell”) increased significantly (from 56.7 to 80.0% (*p* = 0.006) and from 28.3 to 63.3% (*p* = 0.000), respectively) (Table 2).

Table 3, showing the results for 1 March 2020, and Table 4, showing the results for 13 July 2020, show the frequency of the occurrence of recommendations relating to the prevention of COVID-19 provided by the WHO by the type of author and country of origin. They show how the associations between these variables have changed over time. Thus, the weblinks of official public health organizations, compared to those of digital mass media, have always more frequently provided advice to “seek medical advice if you develop a fever/cough or have difficulty breathing”. Furthermore, in July, the weblinks of such organizations more frequently provided advice to “cover your mouth and nose when you cough or sneeze” (OR = 11.5) and “maintain a distance of at least 1 m (3 feet) between yourself and others” (OR = 10.5).

According to the country of origin of the weblink, in March, finding the recommendation to “maintain a distance of at least 1 m between yourself and others” was associated with weblinks made in countries with local transmission/imported cases (OR = 8.14), while in July, the recommendation to “seek medical advice if you develop a fever/cough or have difficulty breathing” was associated with weblinks made in countries with community transmission (OR = 8.85).

In 16 weblinks found in July (15 of them produced in Spain), it was indicated that a safe distance to be maintained from other people is at least 1.5 or 2 m.

Different or ambiguous information regarding the basic prevention measures recommended by the WHO was detected. Thus, a July weblink recommended that people “keep a distance of at least two meters only from people who suffer respiratory infection symptoms”; another July weblink recommended that people “maintain a distance of two meters from other people only if they have a cough or fever”. In March, a weblink specified that “those with acute respiratory infections should maintain their distance from other people”. In addition, a weblink recommended the use of a necktie be temporarily abandoned as a measure to prevent COVID-19.

In the July search, on the first page of results, and located before the first weblink, it was observed how Google had inserted a message of general interest about the prevention measures that were the object of our study, although they only indicated the need to maintain a safe distance from people who cough or sneeze. However, this information was linked to a WHO link, which specified maintaining at least 1 m (3 feet) distance from other people.

## 4. Discussion

This study is the first to evaluate the evolution of the spread of the basic prevention measures of COVID-19 recommended by the WHO available in Spanish on the Internet. “Frequent hand washing” was the main preventive measure found in the weblinks of both moments of the study (93.3–90.0%). In addition, the availability of information on the Internet about two of the basic protective measures (“avoid touching the eyes, nose, and mouth”, and “stay at home if you feel unwell”) increased significantly over time, with increases of up to 35 percentage points.

However, as of July 2020, information on three of the six basic prevention measures (“maintain a distance of at least 1 m”, “if you develop fever/cough or have difficulty breathing, seek medical advice”, and “stay at home if you feel unwell”) was available in less than 67% of the weblinks. This shows that it is still difficult to find such information online, and it represents a worrying finding, since it is very difficult to control the spread of a virus if people have little information about how it can be prevented. Moreover, these results are consistent with what other authors have said regarding the difficulty of finding WHO-promoted measures to prevent other pandemics on the Internet [26]. Thus, Covolo et al., when analyzing the information online about the influenza pandemic vaccine, observed how only 53.9% (41/76) and 80.3% (61/76) of the weblinks provided information on contraindications and indications relating to the vaccine according to the WHO guidelines, respectively [26].

Over time, a change in the main authorship of weblinks was observed, from digital mass media (45.0% of weblinks in March) to official public health organizations (41.7% of weblinks in July). These results could show how official public health organizations were slow to create content or how in March users were subject to SEO strategies that prioritized the mass media and their location strategies in the main search results. In any case, this evolution represents a positive finding, given the reliability of the information provided by official public health institutions [26,30]. In fact, in July 2020, the weblinks of such official public health organizations provided information on three of the basic prevention measures for COVID-19 with a significantly greater frequency than those produced by digital mass media. For this reason, Spanish-speaking users should be encouraged to consult weblinks produced by official public health organizations when looking for information on the Internet on how to prevent COVID-19. The implementation and evaluation of the effectiveness of such measures could be the subject of future research. Additionally, digital mass media must take responsibility for providing correct and complete information and improving the knowledge of citizens [32].

On the other hand, in March 2020, weblinks originating in countries with cases (due to local transmission or imported cases) provided information on “maintaining a distance of at least 1 m between yourself and others” more often than weblinks originating in countries without cases of COVID-19. This could show the effects of the late dissemination of information on basic prevention measures. This would represent a lesson to be learned regarding the management of future public health emergencies of international concern. Regardless of whether there are cases in a country or not, information on how to prevent a disease should be disseminated as quickly as possible, because timely information is a key element of any prevention policy [33], and because the knowledge regarding infectious diseases is associated with the level of adherence to control measures, which may limit the transmission of those diseases [34].

The July recommendation to “seek medical advice if you develop a fever/cough or have difficulty breathing” was found most frequently in weblinks of countries with community transmission (compared with weblinks of countries with clusters of cases). This may indicate how when the epidemiological situation improves, the spread of prevention measures decreases.

The finding that the July search on 15 weblinks originating in Spain indicated that the safe distance to be maintained from other people was at least 1.5 or 2 m was considered to be in line with the WHO recommendations, given that these distances are consistent with the minimum distance of 1 m recommended by the WHO. The explanation for these findings is that a distance of at least 1.5 m is the one recommended by the Spanish Ministry of Health [35,36].

Different or ambiguous information regarding the basic prevention measures of the WHO was detected in 3.33% of the weblinks (4/120). This represents a lower percentage than that described by Baltazar et al. (13.9%), who compared the information provided online on COVID-19 with the medical literature available on PubMed [22]. However, the validity of this comparison is limited by the fact that Baltazar et al. analyzed the information available online mainly in English, and because the focus of the analysis of the information in this study was not only on measures to prevent the disease [22]. In any case, our findings may be relevant to the implementation of educational campaigns, as they may allow for the correction of misinformation about COVID-19 in Spanish.

Ambiguous information was also found in the message inserted by Google about COVID-19 (in particular, regarding keeping a safe distance only from people who cough or sneeze). This would show the need for Google to maintain consistency between the information it provides in that message and the prevention measures recommended by the WHO.

To improve health communication during the COVID-19 pandemic, several considerations have been made; among them, fighting against false information is fundamental [37]. False content has the potential to harm the public. In March 2020, in Iran, 700 people died after ingesting alcohol to treat COVID-19 as a result of misinformation circulating online [38]. For this reason, health communicators are called to revise their strategies to respond to inaccuracies, while also picking up on early signals of rumors and prevent them from spreading further. For example, the WHO has launched an initiative called ‘Mythbusters’, where the WHO directly addresses misconceptions and fake information about the prevention of COVID-19 [39].

Our study has several limitations, among which are those typical of infodemiological studies, in which dynamic information, such as that available on the Internet, is evaluated. However, these types of studies are necessary, because, among other things, they provide valuable insights into the health-related behaviors of populations and allow the distribution and determinants of information on the Internet or in a population to be elucidated [20]. Another limitation, in common with other authors who have analyzed the information available on the Internet about other diseases [27,28,40,41,42], derives from the fact that searches were carried out only on Google [27,28,40,41,42]; however, this search engine is the most popular, covering nearly 90% of the total online searches [43]. The high number of links from Spain obtained in the second search could be due to the fact that the IP address had been identified by not having previously deleted the search history and cookies; regardless, this does not invalidate the concerns described here but rather contextualizes them better.

In addition, like other studies [26,44], the search terms were chosen by the authors with the assumption that an Internet Spanish-speaking user would probably use one of them to perform simple searches regarding COVID-19 prevention measures. Moreover, as was the case for other authors, the search was performed from a single country’s IP address [26,44]; this could have influenced finding weblinks mainly from the country of origin of that IP (Spain). For this reason, future research would be necessary to determine whether results similar to those of this study are obtained when the Google search is performed from IPs of other Spanish-speaking countries.

## 5. Conclusions

The availability of information in Spanish on the Internet on basic COVID-19 prevention measures recommended by WHO has improved over time, although there is still room for improvement. In order for Spanish-speaking users to obtain high-quality information more frequently when searching for information on these preventive measures on the Internet, they should be encouraged to view the websites of official public health organizations. In this way, these websites can also improve their accessibility and positioning, since search engines determine the positioning of the weblinks produced by a search according to the frequency with which they are accessed. For future public health emergencies of international concern, information on how to prevent these diseases should be produced and spread online as quickly as possible by official public health organizations, which could help to better control the spread of the disease.

## Figures and Tables

**Table 1:** Authorship and country of origin of the weblinks by the search date.

Authorship and Country of Origin	1 March 2020	13 July 2020	*p* (between Stages)
	(*n* = 60)	(*n* = 60)	
**Country of origin**			
Spain	37 (61.7)	47 (78.3)	0.047
United States of America	10 (16.7)	6 (10.0)	0.285
Peru	3 (5.0)	0 (0)	0.244
Switzerland	2 (3.3)	5 (8.3)	0.439
Cuba	2 (3.3)	0 (0)	0.496
Others	6 (10.0)	2 (3.4)	0.272
**Type of authorship**			
Digital mass media	27 (45.0)	6 (10.0)	0.000
Official public health organizations	14 (23.4)	25 (41.7)	0.033
Libraries	5 (8.3)	2 (3.3)	0.439
Scientific societies	3 (5.0)	2 (3.3)	1.000
Official non-public-health organizations	2 (3.3)	9 (15.0)	0.054
Private health care systems	2 (3.3)	5 (8.4)	0.439
Others	7 (11.7)	11 (18.3)	0.309

**Table 2:** Available recommendations of the WHO by the search date.

Recommendation	1 March 2020	13 July 2020	*p* (between Stages)
	(*n* = 60)	(*n* = 60)	
Wash your hands frequently	56 (93.3)	54 (90.0)	0.509
Cover your mouth and nose when you cough or sneeze	48 (80.0)	51 (85.0)	0.471
Avoid touching the eyes, nose, and mouth	34 (56.7)	48 (80.0)	0.006
Maintain a distance of at least 1 m from others	40 (66.7)	40 (66.7)	1.000
If you develop a fever/cough or have difficulty breathing, seek medical advice	39 (65.0)	36 (60.0)	0.572
Stay at home if you feel unwell	17 (28.3)	38 (63.3)	0.000

**Table 3:** Recommendations relating to the prevention of COVID-19 provided by the WHO and available on the Internet, with associated factors, on 1 March 2020.

Recommendation	Associated Factors	Available	Unavailable	OR (95% CI) ^a^	*p*
		*n* (%)	*n* (%)		
Wash your hands frequently	**Type of authorship**				
	Official public health organizations	13 (23.2)	1 (25.0)	1.63 (0.15–17.24)	1.000
	Others	19 (33.9)	0 (0)	-	0.257
	Digital mass media	24 (42.9)	3 (75.0)	1	
	**Country of origin**				
	Local transmission or imported cases ^b^	48 (85.7)	4 (100)	-	1.000
	No transmission ^c^	8 (14.3)	0 (0)	1	
Cover your mouth and nose when you cough or sneeze	**Type of authorship**				
	Official public health organizations	12 (25.0)	2 (16.7)	3.53 (0.65–19.09)	0.165
	Others	19 (39.6)	0 (0)	-	0.003
	Digital mass media	17 (35.4)	10 (83.3)	1	
	**Country of origin**				
	Local transmission or imported cases ^b^	42 (87.5)	10 (83.3)	1.4 (0.25–7.99)	0.655
	No transmission ^c^	6 (12.5)	2 (16.7)	1	
Maintain a distance of at least 1 m (3 feet) between yourself and others	**Type of authorship**				
	Official public health organizations	10 (25.0)	4 (20.0)	1.47 (0.36–5.95)	0.734
	Others	13 (32.5)	6 (30.0)	1.28 (0.37–4.42)	0.705
	Digital mass media	17 (42.5)	10 (50.0)	1	
	**Country of origin**				
	Local transmission or imported cases ^b^	38 (95.0)	14 (70.0)	8.14 (1.47–45.19)	0.013
	No transmission ^c^	2 (5.0)	6 (30.0)	1	
Avoid touching the eyes, nose, and mouth	**Type of authorship**				
	Official public health organizations	9 (26.5)	5 (19.2)	3.06 (0.79–11.73)	0.101
	Others	15 (44.1)	4 (15.4)	6.38 (1.65–24.63)	0.007
	Digital mass media	10 (29.4)	17 (65.4)	1	
	**Country of origin**				
	Local transmission or imported cases ^b^	31 (91.2)	23 (88.5)	1.35 (0.25–7.29)	1.000
	No transmission ^c^	3 (8.8)	3 (11.5)	1	
If you develop fever/cough or have difficulty breathing, seek medical advice	**Type of authorship**				
	Official public health organizations	11 (28.2)	3 (14.3)	4.58 (1.04–20.24)	0.039
	Others	16 (41.0)	3 (14.3)	6.67 (1.57–28.37)	0.013
	Digital mass media	12 (30.8)	15 (71.4)	1	
	**Country of origin**				
	Local transmission or imported cases ^b^	34 (87.2)	18 (85.7)	1.13 (0.24–5.29)	1.000
	No transmission ^c^	5 (12.8)	3 (14.3)	1	
Stay at home if you feel unwell	**Type of authorship**				
	Official public health organizations	6 (35.3)	8 (18.6)	2.63 (0.65–10.58)	0.174
	Others	5 (29.4)	14 (32.6)	1.25 (0.32–4.89)	0.751
	Digital mass media	6 (35.3)	21 (48.8)	1	
	**Country of origin**				
	Local transmission or imported cases ^b^	15 (88.2)	37 (86.1)	1.22 (0.22–6.72)	1.000
	No transmission ^c^	2 (11.8)	6 (13.9)	1	

^a^ OR (95% CI): Odds Ratio (95% Confidence Interval); ^b^ countries with local transmission or with imported cases only: the United States of America, The United Kingdom, Spain, and Switzerland; ^c^ Countries without cases (no transmission): Argentina, Chile, Cuba, Colombia, and Peru.

**Table 4:** Recommendations relating to the prevention of COVID-19 provided by the WHO and available on the Internet, with associated factors, on 13 July 2020.

Recommendation	Associated Factors	Available	Unavailable	OR (95% CI) ^a^	*p*
		*n* (%)	*n* (%)		
Wash your hands frequently	**Type of authorship**				
	Official public health organizations	24 (44.4)	1 (16.7)	12 (0.87–165.41)	0.088
	Others	26 (48.2)	3 (50.0)	4.33 (0.54–34.55)	0.195
	Digital mass media	4 (7.4)	2 (33.3)	1	
	**Country of origin**				
	Community transmission ^b^	11 (20.4)	0 (0)	-	0.581
	Clusters of cases ^c^	43 (79.6)	6 (100)	1	
Cover your mouth and nose when you cough or sneeze	**Type of authorship**				
	Official public health organizations	23 (45.1)	2 (22.2)	11.5 (1.33–99.33)	0.038
	Others	25 (49.0)	4 (44.5)	6.25 (0.92–42.51)	0.079
	Digital mass media	3 (5.9)	3 (33.3)	1	
	**Country of origin**				
	Community transmission ^b^	11 (21.6)	0 (0)	-	0.189
	Clusters of cases ^c^	40 (78.4)	9 (100)	1	
Maintain a distance of at least 1 m (3 feet) between yourself and others	**Type of authorship**				
	Official public health organizations	21 (52.5)	4 (20.0)	10.5 (1.41–78.06)	0.026
	Others	17 (42.5)	12 (60.0)	2.83 (0.45–18.04)	0.379
	Digital mass media	2 (5.0)	4 (20.0)	1	
	**Country of origin**				
	Community transmission ^b^	9 (22.5)	2 (10.0)	2.61 (0.51–13.45)	0.307
	Clusters of cases ^c^	31 (77.5)	18 (90.0)	1	
Avoid touching eyes, nose, and mouth	**Type of authorship**				
	Official public health organizations	22 (45.8)	3 (25.0)	7.33 (0.98–54.41)	0.069
	Others	23 (47.9)	6 (50.0)	3.83 (0.61–24.02)	0.162
	Digital mass media	3 (6.3)	3 (25.0)	1	
	**Country of origin**				
	Community transmission ^b^	10 (20.8)	1 (8.3)	2.89 (0.33–25.16)	0.435
	Clusters of cases ^c^	38 (79.2)	11 (91.7)	1	
If you develop fever/cough or have difficulty breathing, seek medical advice	**Type of authorship**				
	Official public health organizations	17 (47.2)	8 (33.3)	-	0.004
	Others	19 (52.8)	10 (41.7)	-	0.005
	Digital mass media	0 (0)	6 (25.0)	1	
	**Country of origin**				
	Community transmission ^b^	10 (25.7)	1 (8.0)	8.85 (1.05–74.50)	0.038
	Clusters of cases ^c^	26 (74.3)	23 (92.0)	1	
Stay at home if you feel unwell	**Type of authorship**				
	Official public health organizations	17 (44.7)	8 (36.4)	2.13 (0.35–12.95)	0.638
	Others	18 (47.4)	11 (50.0)	1.64 (0.28–9.58)	0.665
	Digital mass media	3 (7.9)	3 (13.6)	1	
	**Country of origin**				
	Community transmission ^b^	10 (26.3)	1 (4.5)	7.5 (0.89–63.25)	0.079
	Clusters of cases ^c^	28 (73.7)	21 (95.5)	1	

^a^ OR (95% CI): Odds Ratio (95% Confidence Interval); ^b^ countries with community transmission: the United States of America and Switzerland; ^c^ Countries with clusters of cases: Spain and Germany.
